# Contextualising Long Covid: Viral Sequelae, ‘Post-Encephalitis’ Lethargica and the Modern British Healthcare System, c. 1918–1945

**DOI:** 10.1093/shm/hkae052

**Published:** 2024-09-27

**Authors:** Kate McAllister

**Affiliations:** School of History, Philosophy and Digital Humanities, Jessop West, 1 Upper Hanover Street, Sheffield, S3 7RA, UK

**Keywords:** Long Covid, Encephalitis Lethargica, sequelae, healthcare, Britain

## Abstract

In the months after March 2020, people across Britain began to seek medical attention for protracted illness following an infection with coronavirus disease 2019. Through the efforts of patients, these illnesses were eventually gathered into the diagnostic category of ‘Long Covid’ and therefore viewed as viral sequelae, in turn opening up the possibility for medical care and treatment in the British health system. This article adds to such patient-made knowledge of Long Covid through a comparative historical analysis with the problem of ‘Post-Encephalitis’ Lethargica (EL). In the early twentieth century, the viral sequelae of EL were parsed in line with and thus shaped by the binary divisions that were becoming used to structure healthcare in Britain. By telling this story of the past, this article provides a framework to understand if and how such administrative divisions within the National Health Service (NHS) might continue to inform perceptions of and responses to Long Covid in the present.

In March 2020, people across Britain began to seek medical attention for a series of protracted symptoms, ranging from breathlessness, fatigue, brain fog, body aches, skin rashes, anxiety and low mood. These illnesses, they argued, had begun after infection with coronavirus disease 2019 (COVID-19) yet lasted for many months afterwards. Despite this apparent temporal link, members of the medical and scientific professions disagreed about their possible shared aetiology: with some pulling focus towards ‘minuscule clots, [a] lingering virus, or immune abnormalities’, whilst others underlined the role played by the stress of ‘quarantine, isolation and social distancing’, depression, anxiety or post-traumatic stress disorder.^1^ Through the efforts and advocacy of patients, these illnesses were however gathered into the diagnostic category of ‘Long Covid’, viewed as viral sequelae, and tied to dedicated service provision within the National Health Service (NHS).^2^ Adding to such patient-made knowledge, this article seeks to offer historical context for the category of Long Covid through the problem of ‘Post-Encephalitis’ Lethargica (EL). During the early twentieth century, the long-term sequelae of this epidemic viral disease were parsed in line with and thus shaped by the mental/physical, acute/chronic administrative divisions that were becoming used to structure healthcare in Britain. Exploring this history provides us with an analytical framework to understand how and why such divisions might still be shaping our understanding of and responses to Long Covid today.

This article therefore builds from a simple premise: that during the early twentieth century, healthcare in Britain began to be structured in line with binary distinctions between mental and physical, acute and chronic illness. Since then, certain illnesses that have persisted after a virus and which (at least initially) defied clear theories of causation have conflicted with and brought these structures into sharp focus, in turn generating administrative problems. Categories like ‘Post-EL’, ‘Post-Encephalitic Parkinsonism’ (PEP), and arguably now Long Covid, can be viewed as an attempt to resolve such problems by realigning cases experiencing persisting symptoms of fatigue, pain and low mood with these binary concepts and bringing them within existing health provision. By highlighting the binary structures of healthcare in Britain and how they have shaped the perceptions of and responses to illness that lingers following a virus, this article tells a story of the past which has been ‘crafted in the present’ in order to speak to present concerns.^3^

In the early twentieth century, EL was ‘the third of a triad of infectious neurological diseases’ which swept across Europe and beyond.^4^ The ‘harbingers of [this] new malady’ were identified by neurologist Constantin von Economo during the winter of 1916/17, marking the beginning of an epidemic period that would last until the 1940s.^5^ In Vienna, von Economo observed a growing number of patients displaying ‘excessive sleepiness’ and ‘oculomotor problems, such as partial or complete eye muscle paralysis’.^6^ Based on their clinical similarities, these cases were initially confused with poliomyelitis and linked to the unfolding influenza pandemic, however were soon tied to a distinct yet unknown virus.^7^ In England, some of the first cases were identified in early 1918 by a physician and professor of medicine named Arthur Hall working in the northern city of Sheffield. Over the next 30 years, Hall emerged at the forefront of studies into the ‘acute’ stage of this disease as well as the illnesses which lingered afterwards, thus becoming a nationally and internationally recognised expert.^8^ Hall’s work provides a suitable focus for this article, allowing us to trace how and why categories like EL and Post-EL became used to name these illnesses and ‘appear[ed] at a time, in a place’ yet ‘later fade[d] away’.^9^ Hall’s use of these diagnoses was undoubtedly influenced by a range of ‘particular - social, legal, clinical, cultural, familial and psychological configurations’.^10^ This article nonetheless maintains a deliberately narrow perspective to consider if, how and why the emergence of Post-EL as a category was mediated by more administrative concerns. It therefore focusses on Hall’s attempts, and to a lesser extent those of his contemporaries, to navigate the binary concepts that became embedded in the administrative arrangements of healthcare in Britain in the early twentieth century through particular financial resources, bureaucratic processes and legal criteria.^11^

By adopting this administrative perspective, this article seeks to build a detailed contextual understanding of how and why Arthur Hall came to link a particular set of lingering illnesses to EL during the 1920s. For Hall, establishing whether these symptoms were the result of the short-term action of a virus and of permanent lesions in the brain and body allowed him to make rational decisions about the kind of medical treatment patients might need and to channel them towards particular health services. As a growing number of young, hitherto healthy people presented with persisting fatigue, pain and tremor, Hall nonetheless faced questions about whether their condition was directly linked to a viral attack, or to an ensuing psychic reaction. Neither mental or physical, acute or chronic, these cases failed to fit into existing health provisions and generated administrative problems. Through the category of PEP, however, Hall ultimately parsed the sequelae of EL in ways that tied cases to distinct binary arrangements and brought them within existing health provisions. Through such analysis, this article provides a framework for historians to think with, which can be nuanced and used to analyse the modern British health system, but also ask questions about the ways in which we have understood and responded to long-term virus-related illness, across the past and the present.^12^

The first section begins by mapping out the landscape of health provision in Sheffield, which during the early twentieth century entered a period of reform. As local physicians, health officials and administrators devised new financial mechanisms and bureaucratic processes to widen access to healthcare, they also considered if particular kinds of illness could be distinguished based on their mental or physical, acute or chronic nature, and either allocated to voluntary, public and mental institutions.^13^ In recent years, historians have however argued that such divisions were often not maintained in practice, in part due to a focus on ‘coordination’ across local health systems.^14^ As the first section shows, paying close attention to the specific administrative arrangements used to provide medical care and treatment in Sheffield nonetheless reveals how binary distinctions began to be embedded through distinct financial resources, bureaucratic processes and legal criteria, in turn shaping how Hall would perceive EL.

This argument is explored further in the second and third sections, through a focus on the work of Arthur Hall, the sequelae of EL and the category of PEP. In the early 1920s, physicians across Britain identified a growing number of people who seemed unable to return to school, work or their household duties after EL due to persisting illness. Gathering clinical and statistical data allowed Hall to establish causation and therefore make prognostic predictions and rational decisions about continuing care in many cases. Amongst them however were a group whose fluctuating tremor, fatigue and apathy could not be conceived as mental or physical, acute or chronic, who failed to fit within existing provisions and thus generated administrative problems.

As we shall see in the third section, through the category of PEP Hall offered an alternative model of care and a solution to such problems. Whilst still maintaining the role of short-term viral action and physical damage, Hall highlighted how this condition was also informed by psychic factors. Through this theory of causation, he underlined the need for health provision which rested on intermittent outpatient care at the voluntary hospital. Now intertwined with provisions used to treat acute physical disease, the category of PEP allowed cases to be brought back into the binary administrative structures which were becoming embedded in the health system. Close attention to Hall’s work on the sequelae of EL therefore reveals how healthcare in Britain became organised around the binary concepts of mental/physical, acute/chronic during the early twentieth century and arguably remains so today. As this article will show, such historical context in turn equips us with a framework to both critique the concepts that help us to ‘make sense of (a very small part of) our world’, but also to reflect on how they might be shaping the problem of Long Covid.^15^

## Healthcare in Interwar Sheffield

When the first cases of EL were identified in late 1918, healthcare in Britain was entering into a period of reform. Over the next 30 years, this system would stop being funded via charity and philanthropy and accessible only to a narrow section of the population, and instead become underwritten by a ‘communitarian ethos’ that viewed medical care ‘as a right of citizenship’ and lasts today in the NHS.^16^ This section argues that in interwar Sheffield, such reforms also led physicians to reorganise healthcare in line with the binary concepts of mental/physical, acute/chronic. Though clear institutional divisions did not always hold up in practice, paying close attention to the distinct administrative arrangements used to allocate medical care and treatment reveals how such binary concepts began to be embedded in healthcare during this period. This point is explored further in the next section, through a focus on how and why these concepts framed Hall’s attempts to give meaning and respond to the sequelae of EL.

By the early twentieth century, Sheffield had become a powerful industrial city that contributed centrally to ‘England’s economic development’ and had a growing and politically active population.^17^ As appreciated by local leaders, such economic and industrial output also relied on extensive health services which could ‘cover the entire population of the city and its region’, and which therefore required reform to ensure administrative efficiency.^18^ At the centre of such ensuing health reforms was a Sheffield-born physician named Arthur Hall.^19^ After completing his clinical training at St. Bartholomew’s Hospital in London, Hall returned to his hometown in 1890 to become a physician to the Sheffield Royal Hospital (SRH). He would remain there for the next 40 years, whilst also acting as visiting physician to the South Yorkshire ‘Asylum’/‘Mental Hospital’ and professor of medicine at the University of Sheffield, maintaining his own private practice and sitting on a range of advisory committees.^20^ Straddling various professional and institutional boundaries allowed Hall to take an active role in healthcare reorganisation. In part, such reforms manifested in efforts to align different institutions with particular kinds of mental/physical, acute/chronic illness and disease, as observed by Brian Abel-Smith in his influential study of hospital provision across England and Wales.^21^ Access to medical care and treatment was therefore no longer to be determined by the ability of an individual to pay, and thus mediated by social class, but instead judged in line with more medico-scientific evidence concerning the nature of their condition. With such reforms encouraged by the national Ministry of Health, by the late 1920s Hall and his colleagues in Sheffield had come to agree with the principle that ‘as far as possible acute cases [should] be sent to the voluntary hospitals and the chronic cases to the public hospitals’.^22^

There were four main ‘voluntary’ hospitals in Sheffield: the SRH, the Sheffield Royal Infirmary (SRI), the Jessop Hospital and the Edgar Allen Institute. Prior to the early 1920s, these hospitals were funded primarily via charitable donations, subscriptions or individual ‘private’ payments. This however changed with the introduction of the ‘Penny-In-The-Pound’ (PITP) scheme in 1922, which intertwined access to a short-term or ‘acute’ form of care and treatment with regular fixed wage contributions, made by working men and women.^23^ Undoubtedly generating additional resources, this scheme also ensured that care and treatment within the voluntary hospital was no longer restricted to ‘suitable and deserving cases’ who relied on charity, in turn stimulating additional demand from the working and middle classes.^24^ Whilst access to such care was thus now theoretically judged in line with medico-scientific evidence, these decisions arguably in practice became mediated by work capacity, with the ‘act of contribution’ used to assess and define ‘entitlement to [acute] treatment’.^25^

Whilst such reforms tied voluntary hospitals like the SRH to the treatment of acute illness, more prolonged, chronic forms of care were in contrast to be provided in ‘public’ institutions such as Poor Law infirmaries at Nether Edge in Ecclesall and Fir Vale. These infirmaries were overseen by the local Board of Guardians and funded by ‘rates’ levied by the council via the property tax, however individuals and their families were also charged if deemed appropriate by an almoner or relieving officer.^26^ Admission was in turn tied to the ‘principle of less eligibility’, and reflected the desire to ensure that public relief was ‘less eligible to the recipient than the minimum subsistence they could obtain without’.^27^ In practice, the ‘chronic’ forms of care delivered within these institutions were often taken up by the ‘sick poor’ and the elderly, and were mediated by the ‘stigma of poverty’.^28^

As ‘physical’ forms of illness and disease were thus understood as suitably managed across various public and voluntary hospitals, the care and treatment of insanity generally remained in the legal and institutional context of the South Yorkshire Asylum, or as it was commonly known, Wadsley. Until the late 1920s, care and treatment in Wadsley rested on legal ‘certification’, established to ‘impose coercive control over certain behaviours which were transgressory without amounting to infractions of the criminal law’.^29^ As argued by David Wright, whilst certification undoubtedly imposed a ‘social stigma’ on an individual, this process also had ‘important financial and administrative implications’ as asylum treatment was funded by and expensive for local authorities.^30^ To source the medical evidence needed to establish if an individual was ‘*non compos mentis*’ and therefore suitable for certification, physicians relied on the testimony and ‘influence of family members’ who provided information about how their symptoms had emerged and progressed and how long they had lasted.^31^

Whilst Hall and his colleagues agreed in principle that aligning certain institutions with the binary concepts of mental or physical, acute or chronic could ensure the efficiency which was central to modern healthcare, they also acknowledged that any ‘hard and fast classification’ between different hospitals would be ‘undesirable’ and ‘prejudicial to the interests of patients’.^32^ This reluctance arguably reflects the tension between efforts to embed rigid binary structures and maximise efficiency, within a system facing new dynamics of entitlement and demand, rising costs, yet limited resources and space.^33^ Following the end of the First World War, a ‘spirit of wholesale reconstruction’ had placed further pressure on voluntary hospitals like the SRH, which were increasingly ‘burdened with long waiting lists, but barely had the funds to maintain the existing level of services’.^34^

To relieve such pressure on the voluntary hospitals, the Joint Hospitals Council devised a scheme to spread medical care between these institutions and the Poor Law infirmaries otherwise used to house the chronically sick, with 100 acute beds provided at the ‘Ecclesall Hospital’ at Nether Edge.^35^ There were also moves to facilitate mental and physical forms of care in the SRH via a jointly run ‘mental outpatient clinic’ which in 1927 ‘formally associate[d]’ this institution with the ‘South Yorkshire Mental Hospital’.^36^ Predating the changes in policy implemented via the 1930 Mental Treatment Act, this clinic was intended to facilitate ‘the early treatment of many preventable cases of mental disorder’ and avoid ‘confinement in a mental hospital’.^37^ Such examples of integration during the early twentieth century have been observed elsewhere in the country. As, respectively, shown by Martin Gorsky and Alistair Ritch, Poor Law institutions often provided acute care for the sick poor and also admitted ‘mental’ patients, thus breaching ‘traditional assumptions that general acute medicine was the province of the voluntary sector’.^38^

Although these practical challenges might have led Hall and his colleagues to avoid making hard and fast binary divisions between institutions, these concepts still aligned with distinct administrative arrangements. To receive acute care in the ‘Ecclesall Hospital’ at Nether Edge, for example, patients were not assessed in line with the principle of ‘less eligibility’, but based on their need for ‘hospital treatment’.^39^ Moreover, they did not have to provide evidence of their ‘circumstances and family concerns’ or be subjected to a means test, as long as they could contribute 24s 6d a week towards the cost of maintenance.^40^ In administrative terms, such provision was therefore delivered as in the voluntary hospital, with patients moreover assessed and admitted through a similar bureaucratic process and cared for using acute resources. Though provided within the same institution, this care differed from that provided on chronic wards to patients admitted in line with the principle of less eligibility who relied on the rates. By paying close attention to such arrangements, it thus becomes possible to see how healthcare in Sheffield began to be reorganised in line with binary structures as part of a drive for administrative efficiency during the interwar period. In the next sections, this argument will be explored further through a focus on the work of Arthur Hall, as we explore if and how these structures shaped his perception of EL and its sequelae.

## Arthur Hall and the Sequelae of EL

In 1926, the Medical Research Council (MRC) published the findings of an 18-month study led by Arthur Hall in Sheffield, which had aimed to build ‘knowledge of the distressing mental and physical after-effects of epidemic encephalitis [EL]’.^41^ For the last 4 years, these illnesses had been observed in people across the country, often impacting their ability to work and thus posing a significant economic problem. By correlating the severity of an initial attack of EL to persisting sequelae, Hall and his colleagues provided a clinical framework to help establish if these illnesses were best understood as mental or physical and if they were likely to be chronic, and to position them within health provisions which aligned with these concepts. As we shall see, this framework nonetheless conflicted with cases whose persisting symptoms of fatigue, pain and tremor seemed neither mental or physical, acute or chronic, and who thus presented an administrative problem.

Hall had his first experiences with EL in early 1918, when he was tasked with diagnosing and treating patients admitted to the SRH ‘presenting somewhat remarkable features’.^42^ These patients seemed ‘languid and drowsy’, were ‘unable to move a muscle’ and displayed unusual eye movements such as ‘ptosis’, ‘ophthalmoplegia’ or nystagmus as well as altered speech and muscular tremors.^43^ Though these bodily signs seemed indicative of a ‘lesion’, Hall noted there was no evidence of the ‘usual causes’, such as exposure to cold, trauma, ear disease, rheumatism or syphilis.^44^ Throat swabs had also come back with negative results and there were no visible abnormalities in samples of cerebrospinal fluid. Hall thus vaguely hypothesised that these patients were the victims of ‘something which acts as a widespread poison upon the central nervous system’.^45^ In the coming months, cases were reported by physicians in 51 sanitary districts across England and Wales, in turn reaching ‘double figures’ in Sheffield, London, Birmingham and Leicester.^46^ Based on detailed clinical, epidemiological and pathological investigations commissioned by the Local Government Board, by the end of 1918 this condition was distinguished from cerebrospinal fever and poliomyelitis, linked to an unknown virus and made a notifiable public health problem, hitherto referred to as ‘Encephalitis Lethargica’.^47^ Throughout the 30-year epidemic period, reported cases of this disease would remain low in comparison to other infectious diseases, however numbers rose each winter between 1918 and 1921, reaching a peak in 1924, and were linked to a mortality rate which ‘stubbornly hovered between 35% and 50%’.^48^ Earlier experiences of polio and syphilis moreover raised fears that this virus could have lasting consequences for the health of individuals and in turn the future of the nation. For a country reeling from the effects of war and navigating recurring waves of infectious disease, EL therefore posed a significant additional problem.

In the early 1920s, physicians and school medical officers across Britain began to report persisting illnesses following EL in people of all ages, ranging from lethargy or fatigue, tremor and muscular rigidity or complete behaviour changes.^49^ Still lacking evidence of a common virus, physicians would instead seek to establish causation by gathering clinical information concerning the personal and family history of individual cases and testimony from their parents or friends, husbands or wives, teachers or employers. This information was in turn used to ascertain whether persisting ‘irritability of temper’ or symptoms of ‘paralysis agitans’ could be understood as the result of ‘inflammatory conditions’ caused by a virus even if the initial illness had been ‘abortive’, therefore warranting a diagnosis of ‘Post-Encephalitis’.^50^ As reported by the Ministry of Health, of 3,558 cases notified between 1919 and 1926, a total of 1,464 (41.1 per cent) displayed the ‘sequels’ of EL, leading them to conclude that ‘in proportion to the comparatively small number attacked, there is no infectious or contagious disease… that produces so much consequent ill-health and disablement as does encephalitis lethargica’.^51^

Already recognised for his studies of and expertise on acute EL, Arthur Hall emerged at the forefront of efforts to tackle the long-term effects of this disease. Reflecting on the progress of patients admitted to the SRH, in 1922 Hall acknowledged that whilst many had fully recovered since their acute illness, as evidenced in one ‘old lady’ who was ‘as well as she ever was’, others complained of persisting pain in places such as their head and back.^52^ Over the next year, these illnesses extended to include ‘mental’, ‘respiratory’ and ‘excitomotor residua’, ‘moral changes’, ‘nocturnal excitement’ as well as ‘parkinsonism’.^53^ From the outset, Hall expressed uncertainty about what precisely caused these illnesses, acknowledging that some patients were motivated by the potential ‘receipt of a pension’.^54^ Even in the absence of pathological abnormalities in the cerebrospinal fluid or evidence of the virus itself to suggest an aetiology similar to general paralysis/neurosyphilis, he nonetheless accepted that some patients may still be the victims of the permanent bodily damage. How best to identify these cases and ensure they received appropriate medical care and treatment remained an issue of debate.

Over the next 3 years, as a growing number of patients presented at the SRH to seek ongoing medical care and treatment, Hall gathered clinical information about EL in his case notes.^55^ In the process, he mapped out detailed chronologies which showed how, and in turn aimed to explain why, this disease evolved and persisted over time. To historian Kenton Kroker, through such close attention to his patients and their condition Hall aimed to create an empathetic and therefore efficient system of healthcare.^56^ Such interest in efficiency also arguably informed his efforts to align these illnesses with the binary concepts of mental/physical, acute/chronic. This is reflected in his study of the ‘after-effects’ of EL between 1924 and 1926, for which he received the support of the Medical Research Council and Ministry of Health. As ‘Chairman, or Presiding Genius, or Thruster-in-Chief’, Hall focussed the study on ascertaining in ‘what percentage recovery ha[d] occurred, what kinds of sequelae predominate[d]… whether there [was] any correlation between type of onset and the resulting sequelae’, and understanding the level of ‘economic damage caused by a wave of encephalitis in the community’.^57^ The study played out in two parts: with an initial questionnaire distributed to ‘medical men’ across Sheffield and then followed up with another a year later.^58^ By asking doctors to provide clinical information about the condition of their patient(s), these questionnaires helped Hall and his colleagues to plot out the timeline of their illnesses, to identify patterns in how they emerged and changed over time and to separate them out based on severity.

Overall, 301 cases were studied. Those whose condition was judged as ‘severe’ in its acute stage, based on the testimony of the doctor and often on admission to the hospital, emerged as more likely to develop long-term illness linked to permanent bodily damage caused by EL. In contrast, evidence of an apparently ‘mild’ initial attack could be used to underline the likelihood of recovery and therefore to sever this connection, instead opening up questions about the role played by another medical condition such as hysteria or neurasthenia, or perhaps about the honesty and intentions of the patient and the possibility of malingering. Hall’s use of ‘mild’ and ‘severe’ in this context arguably illustrates a point made by Felicity Callard, about how these concepts become ‘used by different actors, in different locations, in different contexts’, yet often serve to adjudicate symptoms, assess healthcare needs and a ‘return to functional normality, whether inside and outside the labour market’.^59^ In this context, distinguishing cases based on the severity of their condition in the past and present allowed Hall and his colleagues to routinely establish if and when a persisting illness was causally linked to EL. From here, they could then establish whether cases were best understood as mental or physical and if their condition was likely to be chronic, to in turn make decisions about diagnosis and treatment and position them inside or outside of local health provision.

Whilst the findings of this study provided the clinical rationale needed to direct some cases towards institutions that supported long-term, inpatient care for an illness understood as chronic, such as the Poor Law infirmary, it also marked out others who defied this explanatory framework and therefore raised administrative problems. Following an illness categorised as ‘mild’, cases within this group had been forced to step back from their jobs and daily responsibilities due to ongoing fatigue, tremor, muscular rigidity and pain. Many reported that they had lost interest in activities which they previously enjoyed and felt a general apathy towards life. What Hall found ‘particularly surprising’, was that this condition seemed most common in patients aged between 15 and 35 rather than those in their ‘declining years’, who were expected to be more ‘vulnerable’ to long-term effects and who were often sent to receive chronic care in the Poor Law Infirmary.^60^ There was also a ‘preponderance of the male sex’ who represented 72 per cent of cases.^61^ Some could no longer work, as was the case for one man who lacked the ‘sureness of eye and arm’ needed for his job as a cutler, and for a musician who struggled to ‘keep up with the right hand at the same pace as the left’.^62^ These people were the central targets of healthcare reforms such as the PITP scheme, and whose condition, therefore, undermined efforts to ‘build the healthy [and productive] city’.^63^

Hall would draw this group into sharper focus through his case notes. Whilst he later acknowledged the striking clinical similarities between these cases and those with a diagnosis of ‘paralysis agitans’, a condition long understood to be caused by permanent lesions or bodily damage, they were easily (and often) mistaken for ‘neurasthenia’ due to the fluctuation in their symptoms.^64^ Since the late nineteenth century, the ‘borderland’ category of neurasthenia had been used to reference ‘fatiguability, both psychical and physical’ or ‘nervous weakness’, directly triggered by ‘behavioural factors such as overwork, poor diet, lack of exercise, sexual excess, or emotional strain’ but also linked to heredity, a chemical imbalance, or as Mark Honigsbaum has shown, a viral infection.^65^ Neurasthenia was treated through rest, exercise, ‘tonics’ or electricity to ‘restore depleted energy and to control unruly bodies’, often provided as part of ‘convalescent’ care.^66^

In interwar Sheffield, convalescent care was delivered on one of two sites and included in the PITP scheme, thus playing an ‘auxiliary role for the voluntary hospitals in freeing “blocked” beds’.^67^ As with acute forms of care, during this period convalescent care was mediated by new dynamics of entitlement, as it became a benefit earned through contributions to aid recovery from an acute illness or surgical procedure and a return to normal working life.^68^ As shown by Sally Sheard, this shift ‘created tensions in the allocation of funds between hospitals and these additional services’.^69^ Rising demand and limited resources thus warranted mechanisms of gatekeeping in order to reduce pressure on these interconnected institutions, through rigid criteria for admission to convalescent provision which excluded those ‘of too chronic or incurable character’.^70^

Perhaps mindful of these practical challenges and criteria for admission, Hall believed that in spite of the clinical similarities between neurasthenics and certain cases with a history of EL, they were not suited to the same kind of treatment. Through his studies, Hall had meticulously surfaced signs of permanent lesions caused by a virus in the bodies and brains of these cases: recording information about the fixity of their stare, the level of grease on their face or the shakiness of their movements, and tracing if and how this changed over time. Relying on his expert clinical judgement, Hall insisted that these bodily signs were indicative of permanent physical damage, that they would likely endure for long periods of time and thus could not be treated as neurasthenia. These clinical signs were therefore important clues concerning the physical causes and chronic prognosis of this condition.

Whilst Hall might have been convinced that these cases required a form of long-term medical intervention that was beyond the remit of convalescent care, there were also problems associated with providing this in other existing provisions. Many of these cases were ‘profoundly depressed and melancholic’, appeared ‘quite apathetic’ and sometimes ‘attempted suicide’, thus raising the possibility that they might be suited for admission to Wadsley.^71^ This procedure of admission was not uncommon: as reported by the national Board of Control in 1924, 116 cases with a history of EL had been admitted to ‘mental institutions’ across England and Wales since the beginning of the epidemic, due to symptoms of ‘restlessness and excitement’ and in some cases, melancholy and apathy.^72^ In gathering information about their lives prior to EL, Hall had however come to believe that such behaviours were the effects of a virus which had caused an almost complete change in character. This belief was reinforced by family members who arguably resisted the suggestion that their loved one was ‘insane’, leaving Hall without the medical evidence needed to validate certification and their admission to Wadsley and thus understand their condition as mental.^73^

Similarly, whilst the Poor Law infirmaries at Nether Edge and Fir Vale might have been the most practically appropriate space for ‘chronic’ care and treatment, they were often used to house the elderly and sick poor and required the use of a means test to judge eligibility. In contrast, many of the patients experiencing persisting fatigue, pain, tremor and apathy were young, hitherto ‘productive’ men and women, who contributed to the PITP scheme and were ‘good citizens’, in turn establishing their entitlement to a particular programme of acute medical care and treatment.^74^ Hall’s inability to find a place for these cases within existing health provisions can therefore be understood to reflect his commitment to developing and maintaining an efficient system, but also to his patients and their expressed preferences, which together left him unable to establish whether their condition was mental or physical, acute or chronic.

For Hall, these patients posed an administrative problem. Though they were clearly no longer suited to receive acute treatment, provided on an inpatient basis in the SRH or in the wards of ‘Ecclesall Hospital’, or on an outpatient basis in the mental disease clinic, they were equally unsuited for admission to an institution better equipped to deal with a more prolonged, chronic form of care provided in a Poor Law infirmary. Whilst the characteristics of their condition may have also seemed somewhat ‘mental’, many of these patients fell outside of the legal criteria and bureaucratic procedures used to guide admission to an asylum. Paying attention to the problems generated by these patients not only helps us to understand why Hall and his colleagues expressed concern about mapping the binaries of mental or physical, acute or chronic onto different institutions, but also how these concepts shaped their perception of viral sequelae. As the next section will show, in order to resolve such problems Hall would however propose a diagnosis and programme of medical care that fitted into and maintained binary administrative structures, making them visible to the historian.

## Administrative Problems, Medical Solutions

By the mid-1920s, physicians across Britain had begun to acknowledge the trail of ‘after-effects’ associated with EL, as ‘victims of this disease… [were] attending the out-patient department of most hospitals in increasing numbers’.^75^ Amongst them were a group of patients whose condition seemed neither mental or physical, acute or chronic, and which therefore generated administrative problems.^76^ This section explores how Hall offered a possible solution to these problems, via the category of ‘Post-Encephalitic Parkinsonism’.^77^ By using his case notes to identify and arrange significant events in a linear chronology spanning past and present, Hall established that these illnesses were essentially caused by physical changes in the body and brain but also by ensuing psychic responses. With the right kind of treatment and support, these cases might therefore return to the labour market. Intertwining a new model of intermittent yet prolonged outpatient care with a new theory of causation and a new diagnostic category, Hall thus showed why it made sense to pursue a programme of long-term care outside of the Poor Law, which relied on a fixed and pre-existing set of acute arrangements based in the voluntary hospital, and thus aligned with binary administrative structures.

Whilst Hall had seen and treated patients in the acute stages of EL as inpatients at the SRH since 1918, in the mid-1920s this provision expanded to include a dedicated outpatient clinic. In Britain, hospital outpatient services had steadily increased since the eighteenth century in line with rising demand, becoming viewed as an incredibly effective way of ‘sorting and progressing patients through the mass health-care system’.^78^ In contrast to inpatient care, the outpatient clinic had ‘few constraints’ on expansion, required ‘minimal investment in buildings or resources’ and allowed patients to be examined, sorted and only admitted when necessary.^79^ In Sheffield, outpatient provision grew by just under forty percent between 1918 and 1938, meaning that patients could be followed up ‘for longer periods and with less inconvenience’.^80^ For Hall, the EL clinic at the SRH would initially allow him to study this disease and its after-effects, but also to facilitate more long-term forms of treatment and act as a buffer to other institutions.

Although Hall might have kept in contact with some cases since their acute attack, many were referred to his EL clinic by their panel doctor or ‘the representative of a large manufacturing concern’ when it became clear that their illness was persisting.^81^ Upon presentation, Hall began by recording information about their clinical condition, looking to the circumstances of their lives across the past and present and borrowing from physiological theories of adaptation. Such theories were rooted in the work of Herbert Spencer, who in the nineteenth century had defined health, disease, illness and ‘life itself’ as ‘continuous adjustment of internal to external relations’.^82^ Deployed in the 1920s and 1930s by physiologists in Britain and the USA, Hall used theories of adaptation to replot the pathological process that followed upon EL. Fluctuating symptoms of tremor, rigidity and apathy were now tied to continuing maladaptation, which was not only determined by the amount of physical damage produced in the acute viral attack but also by psychic factors such as ‘temperament’ and ‘surroundings’.^83^ Together, these causal factors influenced if and how each individual was able to interact and cope with the world around them after EL.

From this perspective, changes or inconsistencies in symptoms were no longer understood in line with the logics of malingering (and as consciously or unconsciously feigned) but instead as a ‘dynamic response to loss, lack or difficulty’, caused in part by bodily change produced by a virus.^84^ The unique ‘adaptability of youth’ therefore explained why some patients were ‘capable of doing more than one expects’ and could ‘compensate for their partial disability’, whilst others seemed to struggle in comparison.^85^ A ‘fine strong young man’ could conceivably drive a three-ton lorry from Sheffield to London and back in 3 days, yet at other times suffer from tremor, stiffness or paralysis that was linked to EL and warranted a diagnosis of PEP.^86^

Hall reaffirmed this aetiology through trialling particular forms of treatment, such as prescriptions of belladonna.^87^ After giving tinctures to 19 cases in his outpatient clinic, Hall later noted that six had not benefitted at all, three simply ‘claimed’ to have ‘felt better’, yet a further ten displayed improvement that was ‘astonishing’.^88^ Whilst one young man had not washed, dressed or fed himself ‘without assistance’ for several months, after receiving this treatment he had become ‘bright and alert’.^89^ Similar improvement was noted in another case, who had been ‘completely helpless’ yet now did ‘everything for himself, chop[ping] the sticks and help[ing] his mother in household work every day’.^90^ Reflecting on these results, Hall noted the same ‘course of events in many purely psychasthenic cases’, concluding that ‘in all these Parkinsonian cases the “functional” element [was] pronounced’.^91^

Yet, he also maintained that there was ‘no evidence that the actual lesion [was] in any way affected in its condition or progress’ by the use of this medication.^92^ Although belladonna was thus unlikely to heal the ‘very definite organic substratum of the disease’, it could inform a kind of ‘suggestion’ whereby the activity of the mind was redirected away from this damage and the ‘great mass of functional symptoms’ which masked ‘the extent of the real disorder’ lifted, therefore restoring the patient ‘to a life of usefulness’.^93^ Reading against the grain of Hall’s studies might also help us to understand how this perception was shaped by patients themselves. In a letter published by *The Lancet* in 1934, Hall acknowledged how some of his patients increased their daily dosage of belladonna against his advice yet exhibited surprising improvement. Their decisions led Hall to conclude that such improvement had occurred ‘quite independent of suggestion’, and arguably reinforced his belief in the presence of bodily damage.^94^

By underlining the essentially physical nature yet also the psychic element of PEP, Hall showed why cases who received this diagnosis did not necessarily need long-term inpatient care. With help and support, these cases could learn to adapt to their condition, carrying ‘on their ordinary life without any special modification other than is necessary to meet existing disabilities’.^95^ PEP could be treated through medications like belladonna, which were in turn prescribed, monitored and adjusted by expert physicians like himself over long periods of time in outpatient clinics, and managed in line with the resources and provisions associated with acute treatment. This was a category and model of care that made sense practically and financially, which facilitated a return to work and contributed to the costs of the voluntary hospitals through the PITP scheme, yet also aligned with the preferences expressed by patients themselves. In the process, Hall also made space for a hitherto problematic group of illnesses within binary structures and in turn within this local healthcare system.

During the 1920s such administrative problems were faced by physicians across Britain. Whilst these physicians often took different approaches to accommodating patients within local health provisions, such programmes of care still arguably slotted into with binary administrative structures, yet informed different perceptions of viral sequelae. This point can be explored further through turning to the ‘Post-Encephalitis’ section established at Southmead Hospital in Bristol by Percy Phillips.^96^ As acknowledged by a medical officer to the Ministry of Health Allan Chilcott Parsons, ‘special provision’ like the unit at Southmead offered a possible solution to the care of patients who ‘returned home after a prolonged illness in hospital’ and tried to ‘carry on as before’, but became ‘careless and lethargic’, apathetic and forgetful, slow and weak.^97^ Though these patients remained legally ‘uncertifiable for a considerable time’ and therefore unsuitable for a mental hospital, they were equally ‘too chronic and hopeless for hospitals and convalescent homes’, and did not welcome ‘accommodation in the Poor Law Infirmary’, thus raising administrative problems.^98^

As shown by George Gosling, whilst Southmead was a Poor Law institution overseen by the local Board of Guardians, it had been ‘purpose-built as a medical facility’ in 1920 to facilitate ‘the treatment of sick persons of all classes’.^99^ Paying attention to the arrangements, resources and criteria associated with the Post-Encephalitis section reveals the binary distinctions used to separate care within this institution. Whilst the provision of acute treatment at Southmead rested on ‘essentially the same payment system as the voluntary hospitals’ and thus the act of contribution, patients experiencing the persisting effects of EL were admitted after being assessed by the ‘relieving officer’ who looked for evidence of their ‘inability to find employment and their consequent destitution’.^100^ Their admission to the Post-Encephalitis section, therefore, hinged on the traditional eligibility principle that an individual had to be ‘deserving’ and that this could be judged based on their ability to ‘labour for their own bread’.^101^ This evidence of work (in)capacity informed a diagnosis of ‘*chronic* encephalitis lethargica’, as did a referral out of and away from the acute arrangements of the Bristol Royal Infirmary or General Hospital.^102^ Though the perception of and responses to patients with lingering pain, weakness, fatigue and apathy at Southmead was quite different to that of Hall in Sheffield, in both instances the capacity to return to and remain in work was used to judge the course and duration of their condition, and in turn bring them in line with binary administrative structures.

Through a focus on the work of Arthur Hall in Sheffield, this section has argued that the category of PEP came into being during the mid-1920s to bring a particular group of persisting illnesses in line with existing health provisions and binary administrative structures. By the early 1940s, Hall had however begun to turn his attention to the related problem of ‘chronic parkinsonism’, acknowledging the possibility that the ‘activity of the infective agent [did] not necessarily cease when the acute stage [was] over’, and subtly but firmly underlining its physical status.^103^ Though a diagnosis of PEP would remain used by some physicians into the 1960s, this became less common the distance from the end of the epidemic widened.^104^ This gradual disappearance of diagnoses like PEP and ‘chronic encephalitis lethargica’ arguably occurred as the perceptions and responses to viral sequelae began to change, in line with ‘fears about the breakdown or destabilisation of social order’ and broader shifts in how chronic forms of illness were understood.^105^ The subsequent emergence of a ‘psychosocial’ approach encouraged physicians to link certain illnesses to ‘buried emotions’ even if they followed upon and seemed connected to the physical effects of a viral infection, and in turn to draw on a different set of ‘mental’ diagnostic categories such as ‘neurosis’ and modes of psychotherapeutic intervention.^106^

Hall would continue to treat patients in his EL clinic at the SRH until his retirement in the mid-1940s. Although responsibility for some cases was taken on by Dr Raymond Tom Gaunt after this point, the clinic itself disappears from the historical record with the reorganisation and rationalisation of healthcare in Sheffield, as part of the move towards a centrally coordinated, ‘taxpayer-funded, universal and free-at-the-point-of-use’ National Health Service.^107^ Historians like Charles Webster have claimed that by bringing ‘mental and general medicine under the same administrative body’, this new service marked the end of clear binary division within the British health system.^108^ This sense of progress has also been sketched onto changes in how and where chronic forms of illness and disease were managed, which had begun with the dissolution of the Poor Law system, yet was reinforced by the cooperation of hospitals and ‘general practice’ and the shift to care in the community.^109^ Whilst the NHS has become known today for its ‘hierarchised bureaucracy’ and in turn its administrative complexity, the focus amongst policymakers remains on delivering ‘integrated’, equal care across various (e.g. acute, community) services and ensuring ‘parity of esteem’ between mental and physical illness.^110^ Through returning to the problem of Post-EL, this article has however shown that focussing on contemporary perceptions of and responses to viral sequelae might help to grasp if, how and why binary divisions endure within this complex twenty-first-century system. This argument will be explored in the conclusion to this article, in order to understand if and how these structures are shaping our understanding of Long Covid.

## Contextualising Long Covid

In the early twentieth century, healthcare in Britain began to be organised according to the binary concepts of mental/physical, acute/chronic. As observed through a focus on the work of Sheffield-based physician Arthur Hall, these concepts in turn shaped how he understood the long-term fatigue, pain and muscular weakness that followed upon EL. Particularly common in young, previously fit and healthy men, these lingering illnesses seemed neither mental or physical, acute or chronic, and thus generated administrative problems. Through the category of PEP, Hall nonetheless proposed a diagnosis and programme of long-term care which made use of existing acute arrangements available at a local voluntary hospital. Elsewhere in the country, physicians similarly parsed, conceptualised and managed the long-term effects of EL by bringing patients within the administration of the Poor Law and in contrast defining their condition as chronic. By showing how the context-specific diagnosis of PEP emerged in interwar Britain to contain and bring certain viral sequelae within binary structures, this article has aimed to provide an analytical framework to highlight and reflect on the administrative concerns that have perhaps also shaped our understanding of Long Covid.

First used on Twitter by Elisa Perego in May 2020 to summarise her experiences (as well as those of many others) of a ‘longer, more complex course of illness than that emerging from initial, formal reports from Wuhan’, the category of Long Covid was developed by patients in order to ‘intervene ontologically in formulations of COVID-19… by complicating the “biphasic” disease pathway… and pointing to multiple sequelae’.^111^ Due to ongoing debates about causation, patients hoped that a diagnosis of Long Covid would ‘keep aetiological possibilities open’ by side-stepping the temporal markers of ‘chronic’ and ‘post’.^112^ In recognition that these complex, heterogenous illnesses reflected a ‘genuine’ medical condition, in October 2020 the British government allocated specific funds and resources to facilitate the diagnosis and treatment of Long Covid in adults within the NHS, through a nationwide network of ‘multidisciplinary’ clinics.^113^

Since late 2020, Long Covid clinics (or ‘hubs’) have been established across England and Wales. NHS guidance states that a referral should be considered if patients have had symptoms lasting 4 weeks or more following ‘confirmed or suspected COVID-19’, if these symptoms are ‘non-improving’ and ‘having a significant impact on normal activities of daily living’ such as work and care responsibilities, and if ‘further assessment [is] needed to confirm the diagnosis/consider alternative diagnosis’.^114^ Led by ‘a doctor with relevant skills and experience’, these clinics focus on ‘assessing physical and mental health symptoms’ and therefore comprise ‘an integrated pathway of assessment, medical treatment and multifaceted rehabilitation including psychology with direct access to required diagnostics’.^115^ As this article has shown, it is worth taking a closer look at the administrative arrangements associated with such provision, to understand what this integration means in practice, and in turn how this might shape our perception of Long Covid. To do this, we shall briefly return to Sheffield.

In Sheffield, a ‘long’/‘post’-Covid Rehabilitation Hub was established in January 2021 by Sheffield Teaching Hospitals NHS Foundation Trust, after being developed ‘in partnership with primary care, mental health services, patients and researchers’, and was listed as part of acute hospital provision.^116^ With these services delivered and resourced in line with short-term administrative arrangements, this article has shown that we might wish to ask questions about whether they are imposing the kind of fixed course and duration on these illnesses which was originally resisted by patients.^117^ The same might also be true with regard to the criteria and procedures used to facilitate admission and discharge. Patients are referred to the Hub by their general practitioner, who is expected to follow the ‘Sheffield Long Covid Symptom Pathway’.^118^ This pathway states that general practitioners should conduct a preliminary assessment to establish a suspected diagnosis of post-covid, of an acute or life-threatening complication, severe psychiatric symptoms or a risk of self-harm or suicide. Each of these options, in turn, correlates to different bureaucratic processes, which either channel patients towards inpatient admission at an acute hospital, or to the mental health services overseen by the Sheffield Health and Social Care NHS Foundation Trust, where they could receive an ‘urgent psychiatric assessment’.^119^

After requesting a series of pathological tests via the ‘ICE’ system and receiving negative results, general practitioners are to make a referral to the local Hub where patients are offered an hour-long assessment conducted by a specialist therapist. After this, a decision is made about their rehabilitation plan, with options ranging from ‘self-management advice’, enrolment onto a ‘virtual rehabilitation programme’ or a referral to other healthcare and voluntary community providers which include psychological services such as IAPT, to ME/CFS services or the Sheffield Occupational Health Advisory Service (SOHAS). Patients might also be allocated a follow-up appointment at the Hub or discharged if further rehab is deemed unnecessary. Paying attention to such criteria and procedures helps us to begin to see how these services encourage physicians to gather evidence and make decisions to establish if a lingering illness is best understood as mental or physical, acute or chronic, and to use such information to position patients within existing health provision.

As observed in relation to Post-EL, from an administrative perspective, a diagnosis of ‘Long Covid’ might therefore be understood as a way to gather in, contain and manage illnesses in the context of ‘overwhelmed’ services.^120^ Whilst this category emerged through the work and efforts of patients who hoped to leave the aetiology of their condition open, it has arguably become tied to and shaped by services that are presented as ‘holistic’, yet in practice facilitate clear binary division within and across the British health system. Acknowledging this allows us to start pushing back against the way that patients experiencing long-term illness following COVID-19 are understood and dealt with, in the NHS and beyond.

Reaching back in time to Post-EL also helps us to shed light on a possible future for Long Covid. In the third section of this article, we saw how the category of PEP began to shift in line with concern about the financial and material cost of chronic illness and efforts to reframe and deal with many as psychological/mental problems, with symptoms of tremor, fatigue or apathy reinterpreted as the hallmarks of mental conditions such as neurosis, anxiety or depression. There are signs that a similar process is unfolding in relation to Long Covid: in the increasing focus of government funding on research ‘that looks at psychological factors, full stop’, interest in the possibility that this condition may be akin to Myalgic Encephalomyelitis and Chronic Fatigue Syndrome (ME/CFS), making it ‘all in one’s head’ and thus worthy of psychological intervention, and a subtle but significant shift towards the category of ‘Post-Covid’.^121^ Exploring links between the present problem of Long Covid and categories now buried deep in the past thus helps to reveal the binary administrative structures of healthcare in Britain, which have long and still continue to shape how we understand and categorise illness. Perhaps more importantly, such analysis equips us with the insight to begin to mark out these structures, and to challenge the ways in which they cut ‘across living bodies and the temporalities in which they live’.^122^

## Footnotes

1 Jennifer Couzin-Frankel, ‘Clues to Long Covid’, *Science*, 2022, 376, 1261–65, 1262; Siu Wa Tang, Brian E. Leonard and Daiga Maret Helmeste, ‘Long Covid, Neuropsychiatric Disorders, Psychotropics, Present and Future’, *Acta Neuropsychiatrica*, 2022, 34, 109–26, 109; See also Michael J. Peluso and Steven G. Deeks, ‘Early Clues Regarding the Pathogenesis of Long Covid’, *Trends in Immunology*, 2022, 43, 268–70; B. D. Kelly and G. Gulati, ‘Long Covid: The Elephant in the Room’, *QJM*, 2022, 115, 5–6.

2 Felicity Callard and Elisa Perego, ‘How and Why Patients Made Long Covid’, *Social Science and Medicine*, 2021, 268, 113426; *Long Covid Patients to Get Help at More Than 60 Clinics*, https://www.england.nhs.uk/2020/12/long-covid-patients-to-get-help-at-more-than-60-clinics/, accessed 22 April 2024.

3 Chris Millard, *A History of Self-harm in Britain* (Basingstoke: Palgrave Macmillan, 2015), 3.

4 Paul Foley, *Encephalitis Lethargica: The Mind and Brain Virus* (New York: Springer, 2018), 58.

5
*Ibid.*, 15.

6
*Ibid*., 15; See Constantin von Economo, ‘Encephalitis Lethargica’, *Wiener Klinische Wochenschrift*, 1917, 30, 581–85.

7 For more on the polio epidemic in Hungary, see Dora Vargha, *Polio Across the Iron Curtain: Hungary’s Cold War with an Epidemic* (Cambridge: Cambridge University Press, 2018). For more on the influenza pandemic, see Mark Honigsbaum, *Living with Enza: The Forgotten Story of Britain and the Great Flu Pandemic* (Basingstoke: Palgrave Macmillan, 2009); Early twentieth-century scientists were never able to identify a common viral agent, informing an aetiological debate that continues today. See Leslie A. Hoffman and Joel Vilensky, ‘Encephalitis Lethargica: 100 Years After the Epidemic’, *Brain*, 2017, 140, 2246–51.

8 Hall’s expert status was confirmed with the publication of his monograph in 1924, which provided a comprehensive account of the clinical and pathological manifestations of ‘epidemic encephalitis’. See Arthur Hall, *Epidemic Encephalitis* (Bristol: John Wright & Sons, 1924).

9 Ian Hacking, *Mad Travellers: Reflections on the Reality of Transient Mental Illness* (Massachusetts: Harvard University Press, 2002), 1.

10 Felicity Callard, ‘Psychiatric Diagnosis: The Indispensability of Ambivalence’, *Journal of Medical Ethics*, 2014, 40, 526–30, 526, 530.

11 This article adopts the definition of ‘administration’ provided by the Cambridge Dictionary. See *Administration*, Cambridge Dictionary, Cambridge University Press & Assessment 2023, https://dictionary.cambridge.org/dictionary/english/administration, accessed 22 April 2024.

12 For example, this analytical framework might be nuanced further through a comparative perspective that explores the structures of healthcare across or within different localities, regions and countries.

13 The language used in this article shifts in with changes made to healthcare in the early to mid-twentieth century. For example, in the 1920s the ‘asylum’ became the ‘mental hospital’ in line with efforts to destigmatise mental illness. Similarly, the transition from charitable to state sources of funding redefined ‘voluntary’ as ‘general’ hospitals.

14 See for example Martin Gorsky, ‘“Threshold of a New Era”: The Development of an Integrated Hospital System in Northeast Scotland, 1900–39’, *Social History of Medicine*, 2004, 17, 247–67, 250.

15 Millard, *A History of Self-harm in Britain*, 11.

16 Gorsky, ‘Threshold of a New Era’, 250.

17 Barry M. Doyle, *The Politics of Hospital Provision in Early Twentieth Century Britain* (Abingdon: Pickering & Chatto, 2016), 12, 32.

18 Steve Sturdy, ‘The Political Economy of Scientific Medicine: Science, Education and the Transformation of Medical Practice in Sheffield, 1890–1922’, *Medical History*, 1992, 36, 125–59, 152.

19 Hall came from a well-known family in Sheffield. He was the son of respected surgeon John A. Hall, who had worked at the Sheffield Royal Infirmary. His brother, T. Walter Hall, was a prominent local historian. See G. H. Brown, ‘Sir Arthur John Hall’, *Royal College of Physicians*, https://history.rcplondon.ac.uk/inspiring-physicians/sir-arthur-john-hall, accessed 3 May 2024; Arthur Gurney Yates, ‘Obituary: Sir Arthur Hall’, *BMJ*, 1951, 1, 140–41.

20 The Vice Chairman and chief administrator of the Joint Hospitals Council for many years was T. Walter Hall, the brother of Arthur Hall.

21 Brian Abel-Smith, *The Hospitals 1800–1948: A Study in Social Administration in England and Wales* (London: Heinemann, 1964), 209.

22 Minutes of Meetings of the Honorary Staff, 3 October 1927, Royal Hospital, Sheffield, NHS16/1/2/2/2, Sheffield City Archives, Sheffield.

23 Sturdy, ‘The Political Economy of Scientific Medicine’, 151; The penny-in-the-pound scheme was an initiative local to Sheffield, which functioned on similar lines to National Health Insurance. See Louise Pearson, *The History of Westfield Health Through the War Years 1939–1945* (Sheffield: Westfield Health, 2009), 3.

24 Doyle, *The Politics of Hospital Provision*, 62.

25 John Mohan, Martin Gorsky and Tim Willis, *Mutualism and Healthcare: British Hospital Contributory Schemes in the Twentieth Century* (Manchester: Manchester University Press, 2006), 9, 64.

26 Doyle, *The Politics of Hospital Provision*, 132.

27 Alysa Levene, ‘Between Less Eligibility and the NHS: The Changing Place of Poor Law Hospitals in England and Wales, 1929–39’, *Twentieth Century British History*, 2009, 20, 322–45, 323.

28
*Ibid.*, 323.

29 Clive Unsworth, ‘Law and Lunacy in Psychiatry’s “Golden Age”’, *Oxford Journal of Legal Studies*, 1993, 13, 479–507, 488.

30 David Wright, ‘The Certification of Insanity’, *History of Psychiatry*, 1998, 9, 267–90, 269.

31
*Ibid.*, 270.

32 Honorary Staff Committee, Minutes of Meeting, 3 October 1927, Royal Hospital, Sheffield, NHS16/1/2/2/2, Sheffield City Archives, Sheffield.

33 These pressures have been, respectively, documented by George Gosling, Martin Gorsky and John Mohan. See George Gosling, *Payment and Philanthropy in British Healthcare* (Manchester: Manchester University Press, 2017), 4; Martin Gorsky and John Mohan, ‘London’s Voluntary Hospitals in the Interwar Period: Growth, Transformation or Crisis?’, *Nonprofit and Voluntary Sector Quarterly*, 2001, 30, 247–75, 248.

34 Sturdy, ‘The Political Economy of Scientific Medicine’, 144.

35 Anon, ‘Relief for the Shortage of Hospital Beds in Sheffield’, *British Medical Journal*, 1920, 2, 93; In 1927, Ecclesall Bierlow became known as Nether Edge Hospital.

36 William Crochley Sampson claimed that he had established ‘the first Hospital Mental Out-patient Department’ at the SRH in the late nineteenth century. See Gilbert Mould, ‘William Crochley Sampson Clapham’, *British Journal of Psychiatry*, 1923, 69, 592–93; Sheffield Royal Hospital, *Ninety-Fifth Annual Report* (Sheffield: Greenup & Thompson, 1928), 9, NHS16/1/3/11, Sheffield City Archives, Sheffield.

37
*Ibid.*, 11.

38 Martin Gorsky, ‘Threshold of a New Era’, 254; Alistair Ritch, *Sickness in the Workhouse: Poor Law Medical Care in Provincial England*, *1834–1914* (Rochester: University of Rochester Press, 2019).

39 Anon, ‘Relief for the Shortage of Hospital Beds in Sheffield’, 93.

40
*Ibid.*, 93.

41 Medical Research Council, *Sheffield Outbreak of Epidemic Encephalitis in 1924: The Report of a Sub-Committee Appointed by the Medical Advisory Committee of the Local Division of the British Medical Association* (London: Her Majesty’s Stationary Office, 1926), 4, FD 4/108, National Archives, London.

42 Arthur Hall, ‘Note on an Epidemic of Toxic Ophthalmoplegia: Associated with Acute Asthenia and Other Nervous Manifestations’, *The Lancet*, 1918, 191, 568–69, 568; Hall was one of a few physicians who used the name ‘epidemic encephalitis’ instead of ‘encephalitis lethargica’. This was because he felt that the latter was misleading, as lethargy was often only a small element of the overall clinical picture.

43
*Ibid.*, 569.

44
*Ibid.*, 569; Though scientists and historians have since paid close attention to the possible links between EL and the concurrent influenza pandemic, Hall was sceptical. In his report on the Sheffield outbreak of EL in 1924, for example, he argued that there was ‘no very obvious connexion’ between these diseases. He did, however, recognise that alongside the war, the influenza pandemic had shaped the ‘epidemic character’ of EL, due to a ‘world-wide lowering of resistance’. See Medical Research Council, *Sheffield Outbreak of Epidemic Encephalitis in 1924*, 17.

45 Hall, ‘Note on an Epidemic of Toxic Ophthalmoplegia’, 569.

46 Local Government Board, *Reports to the Local Government Board on Public Health and Medical Subjects: Report of an Enquiry into an Obscure Disease*, *Encephalitis Lethargica* (London: Her Majesty’s Stationary Office, 1918), 43, K22128, Wellcome Collection, Wellcome Trust, London; In the years after 1917, cases of EL were identified in France, Germany, Austria, New Zealand, Australia, North and South America, Africa and China. See Foley, *Encephalitis Lethargica*, Chapter 3.

47 Local Government Board, *Reports to the Local Government Board on Public Health and Medical Subjects: Report of an Enquiry into an Obscure Disease*, *Encephalitis Lethargica* (London: Her Majesty’s Stationary Office, 1918).

48 Foley, *Encephalitis Lethargica*, 186.

49 These illnesses emerged amidst wider concerns about the psychological impact of war on the population, which had been drawn into focus by conditions like shellshock. Though early studies of EL acknowledged that war might have exhausted and weakened the defense of an individual against the viral infection, most members of the medical profession accepted the primarily viral and physiological view of the acute stage and the illnesses that came afterwards, with the notable exception of Francis Graham Crookshank. See Foley, *Encephalitis Lethargica*, 61–65.

50 George Augustus Auden, ‘Behaviour Changes Supervening Upon Encephalitis in Children’, *The Lancet*, 1922, 200, 901–4; S. A. Kinnier Wilson, ‘Cases of Symptomatic Paralysis Agitans Following Encephalitis Lethargica’, *Journal of the Royal Society of Medicine*, 1920, 13, 65–66; Abortive cases did not display any symptoms during their acute illness, and often did not seek medical attention. The idea that EL might cause ‘abortive’ cases emerged in the first months of the epidemic to explain its apparently sporadic yet increasing incidence. See Local Government Board, *Report of an Enquiry into an Obscure Disease*, *Encephalitis Lethargica*, 27.

51 Allan C. Parsons, *Report of an Inquiry into the After-Histories of Persons Attacked by Encephalitis Lethargica* (London: His Majesty’s Stationary Office, 1928) 14–17, 19, 36, M6619, Wellcome Collection, London.

52 Arthur Hall, ‘Encephalitis Lethargica: Some Clinical Observations on Thirty Cases’, *The Lancet*, 1922, 199, 526–28, 527.

53 Arthur Hall, ‘The Lumleian Lectures (Abridged) on Encephalitis Lethargica (Epidemic Encephalitis)’, *The Lancet*, 1923, 201, 731–40.

54 Hall, ‘Encephalitis Lethargica: Some Clinical Observations’, 527.

55 Hall’s case notes are now held by the Special Collections archive at the University of Sheffield.

56 Kenton Kroker, ‘Encephalitis Lethargica: Last Century’s Long Haulers?’, *CMAJ*, 2021, 193, E1468-51470.

57 Letter from unnamed MRC official to Arthur Hall, 15 April 1924, Encephalitis Lethargica: correspondence; reprints, FD 1/590, National Archives, London; Medical Research Council, *The Sheffield Outbreak of Epidemic Encephalitis in 1924*, 33.

58
*Ibid.*, 69–70.

59 Felicity Callard, ‘Very, Very Mild: Covid-19 Symptoms and Illness Classification’, 8 May 2020, http://somatosphere.net/2020/mild-covid.html/, accessed 22 April 2024.

60 Medical Research Council, *The Sheffield Outbreak of Epidemic Encephalitis in 1924*, 42.

61
*Ibid.*, 59.

62 Arthur Hall, ‘A British Medical Association Lecture on Post-Encephalitic Parkinsonism’, *BMJ*, 1926, 1, 127–30, 128; Though Hall sometimes mentioned that cases had fought in the war, this was not something that he commonly highlighted and thus presumably, thought to be relevant.

63 Barry M. Doyle, ‘Sick or Healthy? The Urban North Between the Wars’, *Northern History*, 2023, 60, 94–116.

64 Hall, ‘A British Medical Association Lecture on Post-Encephalitic Parkinsonism’, 129; ‘The Definition and Treatment of Neurasthenia’, *The Lancet*, 1913, 182, 1557–58, 1558; This confusion between cases with a history of EL and neurasthenics was for example highlighted by Ernest S. Reynolds, a Professor of Clinical Medicine in Manchester. See E. S. Reynolds, ‘Post-Encephalitic Parkinsonism’, *British Medical Journal*, 1926, 1, 219.

65 Tracey Loughran, *Shell-Shock and Medical Culture in First World War Britain* (Cambridge: Cambridge University Press, 2017), 58; Charles Rosenberg, *Explaining Epidemics and Other Studies in the History of Medicine* (Cambridge: Cambridge University Press, 1992), 85–87; See Mark Honigsbaum, ‘The Great Dread: Cultural and Psychological Impacts and Responses to the “Russian Influenza in the United Kingdom, 1889–1893”’, *Social History of Medicine*, 23, 2010, 299–319.

66 Mark Jackson, *The Age of Stress: Science and the Search for Stability* (Oxford: Oxford University Press, 2013), 26.

67 Martin Gorsky, John Mohan and Tim Willis, *Mutualism and Health Care*, 136; There were two convalescent homes opened in Fulwood in quick succession: the Woofindin Convalescent Home in 1901 and the Royal Hospital Annexe in 1908. An additional home, named after (and funded by) industrialist and philanthropist Zachary Merton, was adjoined to the Annexe in 1938.

68 Eli Anders, Between Hospital and Home: English Convalescent Care from Nightingale to the National Health Service (PhD diss., John Hopkins University, 2017), 268.

69 Sally Sheard, ‘Getting Better, Faster: Convalescence and Length of Stay in British and US Hospitals’, in Laurinda Abreu and Sally Sheard, eds, *Hospital Life: Theory and Practice from the Medieval to the Modern* (Oxford: Peter Lang, 2014), 299–330, 313.

70 Honorary Staff Committee, Minutes of Meeting 7 June 1920, Royal Hospital, Sheffield, NHS16/1/2/2/2, Sheffield City Archives, Sheffield.

71 Hall, ‘Post-Encephalitic Parkinsonism’, 128.

72 Parsons, *Report of an Inquiry into the After-Histories of Persons Attacked by Encephalitis Lethargica*, 60–61.

73 Hall, ‘Post-Encephalitic Parkinsonism’, 128; The testimony given by family members was central to Hall’s understanding of Post-EL. This is reflected in both his published work and case notes. See, for example, *Ibid.*, 129.

74 These shifting dynamics of entitlement have been explored by Mohan, Gorsky and Willis, see *Mutualism and Health Care*, Chapter 3.

75 Allen Hancock, ‘Parkinsonism Together with the “Apache Type” in Encephalitis Lethargica’, *British Medical Journal*, 1925, 1, 966.

76 These administrative problems were acknowledged by others. See C. M. Smith, ‘Sequelae of Encephalitis Lethargica: Notes on 128 Cases’, *British Medical Journal*, 1927, 1, 872–73; E. Bramwell, ‘The So-Called Parkinsonian Syndrome with Special Reference to Epidemic Encephalitis’, *Edinburgh Medical Journal*, 1925, 32, 129–41; Wilfred Harris, ‘A Case of Post-Encephalitic Parkinsonian Syndrome Without History of Encephalitis’, *Proceedings of the Royal Society of Medicine*, 1926, 19, 7.

77 Hall’s use of this diagnosis was arguably influenced by international colleagues, such as French physician Jean-René Cruchet. As shown by Olivier Walusinski, Cruchet was also a recognised expert on EL who became interested in ‘post-encephalitic bradykinetic syndrome’ in the mid-1920s. Like Hall, Cruchet highlighted that persisting tremors, ocular paralysis and bradykinesis left many people unable to work. There are also interesting parallels between Britain and France during in this period, in the healthcare reforms made to extend access across the population. A comparative analysis of how the category of PEP emerged, evolved and disappeared in each country might therefore help us to see whether binary structures cut across national contexts. See Olivier Walusinski, ‘René Cruchet (1875–1959), Beyond Encephalitis Lethargica’, *Journal of the History of the Neurosciences*, 2022, 31, 45–63; Timothy B. Smith, ‘The Social Transformation of Hospitals and the Rise of Medical Insurance in France, 1914–1943’, *The Historical Journal*, 1998, 41, 1055–87.

78 Doyle, *The Politics of Hospital Provision*, 74.

79 Keir Waddington, ‘Unsuitable Cases: The Debate Over Outpatient Admissions, the Medical Profession and Late-Victorian London Hospitals’, *Medical History*, 1998, 42, 26–46, 30.

80 Doyle, *The Politics of Hospital Provision*, 74; Arthur Hall, ‘Presidential Address’, *Perspectives in Public Health*, 1929, 50, 133–36, 136.

81 Hall, ‘Post-Encephalitic Parkinsonism’, 129.

82 Herbert Spencer, *The Principles of Psychology* (London: Green and Longmans, 1855), 374.

83 Arthur Hall, ‘The Prognosis and Treatment of Chronic Epidemic Encephalitis’, *The Practitioner*, 1934, 133, 26–36, 31; Temperament was defined in different ways during the early twentieth century, however here this term is understood to refer to the ‘inherent emotional potentialities and kinetic tendencies’ which informed ‘action and reaction, [and] outlook upon life’. See for example, E. Fryer Ballard, ‘The Psychoneurotic Temperament and its Reactions to Military Service’, *Journal of Mental Science*, 1918, 64, 365–77, 365.

84 Stefanos Geroulanos and Todd Meyers, *The Human Body in the Age of Catastrophe: Brittleness*, *Integration*, *Science and the Great War* (Chicago: The University of Chicago Press, 2018), 120.

85 Hall, ‘Post-Encephalitic Parkinsonism’, 128.

86 Hall, *Epidemic Encephalitis*, 117.

87 ‘Belladonna’, or ‘deadly nightshade’ became used for medical purposes in the Victorian period because of its ability to dilate the pupils. Gradually, belladonna also became used to treat convulsions, neuralgia, rheumatism, mania, gout and ‘painful conditions of the nervous system’. See Elizabeth A. Campbell, ‘Don’t say it with nightshades: sentimental botany and the natural history of Atropa Belladonna’, *Victorian Literature and Culture*, 35 (2007), 607–15, 612.

88 Hall, ‘Post-Encephalitic Parkinsonism’, 129.

89
*Ibid.*, 129.

90
*Ibid.*, 129.

91
*Ibid.*, 129.

92
*Ibid.*, 130.

93
*Ibid.*, 130.

94 Arthur Hall, ‘Drugs in Post-Encephalitic Parkinsonism’, *The Lancet*, 1934, 223, 595.

95 Hall, ‘The Prognosis and Treatment of Chronic Epidemic Encephalitis’, 36.

96 For more information on Dr Phillips, see Anon, ‘Obituary: P. Phillips’, *BMJ*, 1968, 4, 710.

97 Allan C. Parsons, ‘Post-Encephalitis and its Problems’, *Proceedings of the Royal Society of Medicine*, 1928, 21, 1307–18, 1315.

98
*Ibid.*, 1315.

99 Gosling, *Payment and Philanthropy*, 66.

100
*Ibid.*, 128; Parsons, *Report of an Inquiry on the After-Histories of Persons Attacked by Encephalitis Lethargica*, 107–8.

101 Levene, ‘Between Less Eligibility and the NHS’, 324.

102 Parsons, *Report of an Inquiry on the After-Histories of Persons Attacked by Encephalitis Lethargica*, 107–8; My emphasis.

103 Arthur Hall, ‘Chronic Parkinsonism: Its Progress and Treatment’, *The Lancet*, 1943, 241, 193–94.

104 See for example MacDonald Critchley, ‘Post-Encephalitic Parkinsonism with Marked Palilalia’, *Proceedings of the Royal Society of Medicine*, 1947, 40, 552; Gilbert Onuaguluchi, ‘Crises in Post-Encephalitic Parkinsonism’, *Brain*, 1961, 84, 395–414.

105 Jackson, *The Age of Stress*, 87.

106 See Mathew Thomson, *Psychological Subjects: Identity, Culture and Health in Twentieth Century Britain* (Oxford: Oxford University Press, 2002), 202.

107 Also born and raised in Sheffield, Gaunt left the SRH to work at the Chesterfield and North Derbyshire Royal Hospital in 1948. Hall died at the age of 84 on 3 January 1951. There is no direct mention of the ‘Encephalitis clinic’ in the first report of the United Sheffield Hospitals in 1949, suggesting that if it did still exist, it had been subsumed within a broader Department. See The United Sheffield Hospitals, *First Report of the Board of Governors* (Sheffield: 1949), 31; Andrew Seaton, *Our NHS: A History of Britain’s Best Loved Institution* (New Haven: Yale University Press, 2023), 21.

108 Millard, *A History of Self-harm*, 30; See C. Webster, ‘Psychiatry and the Early National Health Service: The Role of the Mental Health Standing Advisory Committee’, in H. Freeman and G. E. Berrios, eds, *150 Years of British Psychiatry, 1841–1991* (London: Gaskell, 1991).

109 See George Weisz, *Chronic Disease in the Twentieth Century* (Baltimore: John Hopkins University Press, 2014), 182.

110 Rebecca Irons, ‘Challenging NHS Corporate Mentality: Hospital Management and Bureaucracy in London’s Pandemic’, *Medical Anthropology*, 2024, 43, 205–18, 205; This complexity has also been documented by Geoffrey Rivett, see Geoffrey Rivett, ‘2008–2017: An Uncertain Path Ahead’, The History of the NHS, https://www.nuffieldtrust.org.uk/chapter/2008-2017-an-uncertain-path-ahead, accessed 30 April 2024.

111 Callard and Perego, ‘How and Why Patients Made Long Covid’, 113246.

112
*Ibid.*, 113246.

113 ‘NHS to Offer “Long Covid” sufferers help at specialist centres’, 7 October 2020, https://www.england.nhs.uk/2020/10/nhs-to-offer-long-covid-help/, accessed 10 May 2024.

114 NHS England, ‘Commissioning Guidance for Post Covid Services for Adults, Children and Young People’, 2023, 10–11, https://www.england.nhs.uk/publication/national-commissioning-guidance-for-post-covid-services/, accessed 22 April 2024.

115
*Ibid.*, 7.

116 Ianthe Fowler, ‘Long Covid Rehabilitation Hub Referral Information for GP’s’, 2022, https://www.sheffieldccgportal.co.uk/pathways/post-covid-rehabilitation-hub, accessed 22 April 2024.

117 Sheffield Teaching Hospitals Foundation Trust, ‘About Us’, https://www.sth.nhs.uk/about-us, accessed 22 April 2024; Unlike other hospital services listed on the STHNFT website, there is no information provided about the location of the long covid hub or any contact details.

118 Fowler, ‘Long Covid Rehabilitation Hub Referral Information for GP’s’.

119 Sheffield Health and Social Care Foundation Trust, *Welcome*, https://www.shsc.nhs.uk/, accessed 22 April 2024.

120 Kristin Kay Barker, Owen Whooley, Erin F. Madden, Emily E. Ahrend and Neil R. Green, ‘The Long Tail of and the Tale of Long COVID: Diagnostic Construction and the Management of Ignorance’, *Sociology of Health and Illness*, 2022, 1–19, 15.

121 Baroness Sal Brinton, Debate in the House of Lords on Long Covid, 17 November, 2022, https://hansard.parliament.uk/lords/2022-11-17/debates/E105F485-3B07-4020-AA3A-118870DCF534/LongCovid, accessed 14 June 2023; Eleanor Alexandra Byrne, ‘Understanding Long Covid: Nosology, Social Attitudes and Stigma’, *Brain Behavior and Immunity*, 2022, 99, 17–24, 18.

122 Felicity Callard, ‘Epidemic Time: Thinking from the Sickbed’, *Bulletin of the History of Medicine*, 2020, 94, 727–43, 737.

## Notes

Kate McAllister is an early-career researcher based in Sheffield, UK. She recently completed a PhD at the University of Sheffield. Her PhD was fully funded by the Wellcome Trust and explored the relationship between long-term virus-related illness and the binary structures of the British health system across the twentieth century. Kate is currently developing a postdoctoral project on the history of chronic pain in post-industrial Britain, and a book proposal on Encephalitis Lethargica.

